# Fabrication and Characterization of PLA/PBAT Blends, Blend-Based Nanocomposites, and Their Supercritical Carbon Dioxide-Induced Foams

**DOI:** 10.3390/polym16141971

**Published:** 2024-07-10

**Authors:** Kartik Behera, Chien-Hsing Tsai, Xiang-Bo Liao, Fang-Chyou Chiu

**Affiliations:** 1 Department of Chemical and Materials Engineering, Chang Gung University, Taoyuan 333, Taiwan; b.kartik1991@gmail.com (K.B.); a0970600013@gmail.com (C.-H.T.); brian2006tw@gmail.com (X.-B.L.); 2 Department of General Dentistry, Chang Gung Memorial Hospital, Taoyuan 333, Taiwan; 3 Department of Chemical Engineering, Ming Chi University of Technology, New Taipei City 243, Taiwan

**Keywords:** poly(lactic acid), poly(butylene adipate-co-terephthalate), nanocomposites, supercritical carbon dioxide, foams, physical properties

## Abstract

In this study, a twin-screw extruder was used to fabricate poly(lactic acid) (PLA)/poly(butylene adipate-co-terephthalate) (PBAT) blends and blend-based nanocomposites with carbon nanotube (CNT) or nanocarbon black (CB) as nanofillers. The fabricated samples were subsequently treated with supercritical carbon dioxide (scCO_2_) to fabricate the corresponding foams. Bi-phasic morphology and selective distribution of CNTs or CBs in the PBAT phase were observed in the blends/composites through scanning electron microscopy. After the scCO_2_ treatment, the selective foaming of the PBAT phase in the prepared blends/composites was confirmed. The cellular structure of PBAT phase in scCO_2_-treated blends is similar to the size/shape of PBAT domains in untreated blends or treated neat PBAT foam. The addition of CNTs or CBs in the blends led to a slight reduction in cell size of the foamed PBAT phase, demonstrating CNT/CB-induced cell nucleation. Differential scanning calorimetry (DSC) results showed that CNTs and CBs played as nucleating agents and increased the initial crystallization temperature up to 14 °C compared with neat PBAT for PBAT in different composites during cooling. The scCO_2_ treatment induced the bimodal stability of PBAT crystals in different samples, which melted mainly in two temperature regions in DSC studies. Thermogravimetric analyses revealed that compared with parent blends, the addition of CNTs or CBs increased the temperature at 80 wt.% loss (degradation of PBAT portion) up to 6 °C. The electrical resistivity decreased by more than six orders of magnitude for certain CNT- or CB-added composites compared with the parent blends. The hardness of the blends slightly increased after forming the corresponding composites and then declined after the scCO_2_ treatment.

## 1. Introduction

Poly(lactic acid) (PLA), a crucial bio-based and biodegradable polymer made from renewable sources, has gained great attention during the past two decades because of concerns about resource depletion and environmental pollution caused by petroleum-derived polymers [1,2]. The advantages of high stiffness, excellent transparency, good processability, low toxicity, and bio-compatibility offer PLA a wide range of applications in tissue engineering, medication administration, and food packaging [3,4,5,6]. However, the high cost, low ductility, and intrinsic brittleness of PLA limit its widespread adoption in applications requiring good toughness and lightweight compared with commercial polypropylene and polyethylene [2,6].

Foaming is a useful technique that offers numerous advantages and can decrease the weight of polymeric materials [7]. In contrast to their solid counterparts, microcellular-based plastic foams often possess not only lightweight but also superior impact strength (IS), sound insulation, and heat insulation qualities. As a result, the foams are utilized in numerous industries, such as automotive, packaging, safety equipment, and construction [8,9]. The major challenge for foaming PLA lies in its notably low melt strength [10]. Cell coalescence and collapse readily occur due to the lack of robustness in cell walls during their expansion, resulting in the formation of open cells and an uneven distribution of cell sizes. To address this issue, previous works employed various effective methods, including chain extension, crosslinking, and blending, to enhance the foaming capability of PLA [11]. Blending is a cost-efficient and practically suitable approach for industrial-scale production. However, discovering a renewable method to toughen and maintain the rigidity of PLA as effectively as non-renewable methods remains challenging.

PLA has limited foamability and low toughness, which pose a significant obstacle to its widespread usage. Considerable efforts have been dedicated to improving the toughness of PLA [12,13,14,15,16,17]. Poly(butylene adipate-co-terephthalate) (PBAT) has emerged as one of the suitable choices to blend with PLA due to its favorable combination of biodegradability, high ductility, and impact resistance [18]. Wang et al. [19] investigated PLA/PBAT (60/40) blend, which achieved enhanced elongation at break (EB) (579.9%) and IS (29.6 kJ/m^2^) through the incorporation of multifunctional epoxide as a reactive compatibilizer. Wu et al. [20] fabricated a highly durable PLA/PBAT/ethylenemethyl-acrylate-glycidyl methacrylate (EMA-GMA) (75/10/15) ternary blend by using reactive melt blending. The ternary blend exhibited an impressive notched IS of 61.9 kJ/m^2^, nearly 13 times higher than that of the binary PLA/PBAT blend. The addition of 8 wt.% EMA-GMA increased EB to 320%, resulting in a 54.6% increase compared with the binary blend. Liu et al. [21] examined the effect of blending with PBAT on the crystallization behavior and foamability of PLA. Increasing PBAT content from 0 to 7 wt.% increased the PLA cell density from 2.01 × 10^6^ to 1.74 × 10^7^ cells/cm^3^ and the expansion ratio from 4.87 to 10.94. Research has been conducted to enhance the thermal, electrical, and mechanical properties of polymers/blends by incorporating minimal quantities of nanofillers [22,23,24,25]. Of the fillers investigated, carbon nanofillers have been identified as suitable materials for increasing the properties of polymer matrices. For example, carbon nanotubes (CNTs) have shown the potential to remarkably enhance the rigidity and electrical properties of polymers by forming nanocomposites at low loadings (approximately <3 wt.%) [22,24]. Soni et al. [26] reported that carbon nanofiller-based composites are extensively employed in supercapacitors, solar cells, fuel cells, energy storage systems, the automobile sector, the electronics industry, packaging, and electromagnetic interference shielding applications. Urquijo et al. [27] fabricated PLA/PBAT/CNT composites through melt mixing. The IS of the PLA/PBAT (60/40) blend increased by 155% in the presence of 3 wt.% CNTs. The electrical percolation threshold was achieved with 1.28 wt.% CNTs in the PLA/PBAT blend. These findings highlight the incorporation of CNTs into PLA/PBAT blends, prompting further investigation into enhancing PLA foam properties through similar approaches.

Limited research has been undertaken to enhance the properties of PLA foams. Najafi et al. [28] investigated the impacts of long-chain branched (LCB) structure and adding organoclay on PLA foaming and determined the resultant mechanical properties. The incorporation of clay increased the crystallinity of linear PLA but decreased the crystallinity of LCB-PLA. However, in the supercritical carbon dioxide (scCO_2_)-induced LCB-PLA composite foams, a high clay content increased specific IS, which surpassed that of unfoamed specimens. LCB-PLA with 0.5 wt.% clay showed a 37% increase in specific impact resistance, from 13.9 to 19.1 (kJ m^−2^)/(kg m^−3^). Livi et al. [29] investigated PBAT/imidazolium- and phosphonium-treated clay nanocomposites prepared by melt blending. The fabricated samples were subsequently treated with scCO_2_ to prepare foams. The loading of 2 wt.% of imidazolium-treated clay drastically reduced the cell size from 600 μm to 125 μm. Lee et al. [30] found that blending polyhydroxyalkanoate (PHA) and nano-fibrillated polytetrafluoroethylene (PTFE) with PLA improved the IS of the final foams. The specific IS of both unfoamed and foamed samples increased with higher loadings of PTFE and PHA. Li et al. [31] investigated an efficient and eco-friendly approach utilizing scCO_2_ for foaming to produce PLA/CNT nanocomposite foams. The storage modulus of the composites exhibited a three-order-of-magnitude increase compared with that of pure PLA. PLA/CNT foam with a high volume expansion ratio of 49.6 times was successfully produced at a transition temperature of 121 °C. PBAT nanocomposite foams are less studied. Given the promising results from various studies on enhancing PLA foams, scholars should explore the effects of CNTs on PLA/PBAT blend.

According to the literature, PLA/PBAT blends show potential in various applications of biopolymers. The effects of loading CNTs or carbon nanoblacks (CBs) on different properties of PLA/PBAT blends should be worthy of investigation and comparison. In this report, we used CNTs or CBs as nanofillers to prepare PLA/PBAT blend-based nanocomposites through melt mixing. A compatibilizer was not added to the blends and composites. The prepared samples were treated with scCO_2_ to fabricate corresponding foams with selective foaming in the PBAT phase only. The phase morphology, fillers’ dispersibility, crystallization/melting behavior, thermal stability, tensile properties, hardness, and electrical resistivity of scCO_2_-untreated samples were thoroughly assessed and compared. The developed cellular structure, melting behavior, and hardness of scCO_2_-treated samples were preliminarily characterized. This study presents a viable method to fabricate fully bio-based PLA blend/composite foams with partial cellular structure development for potential use in packaging and bio-medical industries.

## 2. Materials and Methods

### 2.1. Materials and Sample Preparation

PLA (Luminy^®^ L175), with a melt flow rate of 8 g/10 min at 210 °C/2.16 kg, manufactured by Total Corbion PLA (Gorinchem, Netherlands) was used. PBAT (Ecoflex^®^ C1200), purchased from BASF Co., Ltd., Ludwigshafen, Germany, was the blend counterpart for PLA. Multi-walled CNTs (ICT030) were purchased from Golden Innovation Business Co. Taiwan; CNTs have purity >90%, an average diameter of around 60 nm, and a length of 1–30 μm and served as nanofiller for preparing PLA/PBAT blend-based nanocomposites. CBs (VXC-72) manufactured by Cabot Corporation (Boston, MA, USA) with a diameter of around 30 nm were used as another kind of nanofiller for nanocomposite preparation.

Neat components were dried in a vacuum oven at 70 °C for 24 h before being fed into a twin-screw extruder to prepare blend/composite samples according to the formulation listed in Table 1. The twin-screw extruder SHJ-20B (Nanjing Jieya Extrusion Equipment Co., Nanjing, China) was employed, and the mixing temperature was set at 130 °C–210 °C from the feeding zone to the die zone at a screw speed ca. 360 rpm. The extruded and then pelletized samples were followed by injection molding (V4-20SP-G, Multiplas Enterprise Co., Taoyuan, Taiwan) and compression molding (Gotech GT-7014, Gotech, Taichung, Taiwan) to prepare specimens for subsequent characterizations. Specimens with dimensions of 20 mm × 5 mm × 1 mm were used for one-step scCO_2_ batch foaming experiments. The specimens were put into an autoclave, and the temperature/pressure (scCO_2_) was adjusted to 100 °C/4000 psi. After soaking in a scCO_2_ environment for 2 h, the pressure was quickly released (less than 5 s) to normal pressure. The soaked specimens were taken out from the autoclave to room temperature.

### 2.2. Characterization

Scanning electron microscopy (Hitachi S-3000N, Tokyo, Japan; Jeol JSM-7500F, Tokyo, Japan) was used to observe the phase morphology and fillers’ dispersibility of the cryo-fractured section of the prepared samples. The samples were immersed in liquid nitrogen before being fractured. The crystallization/melting behavior of PLA and PBAT in various samples was analyzed using a differential scanning calorimeter (TA DSC Q10, TA Instruments, New Castle, DE, USA). The scCO_2_-untreated (unfoamed) samples were heated to 210 °C (molten state) at 20 °C/min and held for 3 min prior to being cooled to 20 °C at 10 °C/min for crystallization behavior study. The pre-cooled samples were subsequently subjected to 20 °C/min-heated to 210 °C for melting behavior investigation. The scCO_2_-treated samples were directly 20 °C/min-heated from room temperature to 210 °C for melting behavior study without pre-cooling. Thermogravimetric analysis (TGA) experiments were carried out using a TA Q50 analyzer (TA Instruments, New Castle, DE, USA) under a nitrogen environment at a 10 °C/min heating rate. Tensile properties of dumbbell-shaped specimens (following ASTM D638) were measured at a crosshead speed of 10 mm/min by using a Gotech testing machine (AI-3000, Taiwan). The averaged values from five specimens of the same formulation were reported. The surface electrical resistivity of strip specimens was measured using resistivity meters (MCP-T700 and MCP-HT450, Mitsubishi Chemical Co., Tokyo, Japan) in a four-probe method. The measurements were performed four times for each specimen at a working voltage of 500 V and a duration of 10 s, and the average values were reported. Density was determined using a density meter (TWS-153E, MatsuHaku, Taichung, Taiwan). The porosity of the foamed samples was determined using the following equation [32]:

(1) $$Expansion\;ratio=\frac{\rho_{s}}{\rho_{F}}$$

where $\rho_{s}$ is the sample density before foaming, and $\rho_{F}$ is the density of the corresponding foamed sample. Hardness was measured using a GS-702N TYPE D durometer (Teclock, Nagano, Japan).

## 3. Results and Discussion

### 3.1. Phase Morphology of Unfoamed Samples

**Figure 1:**
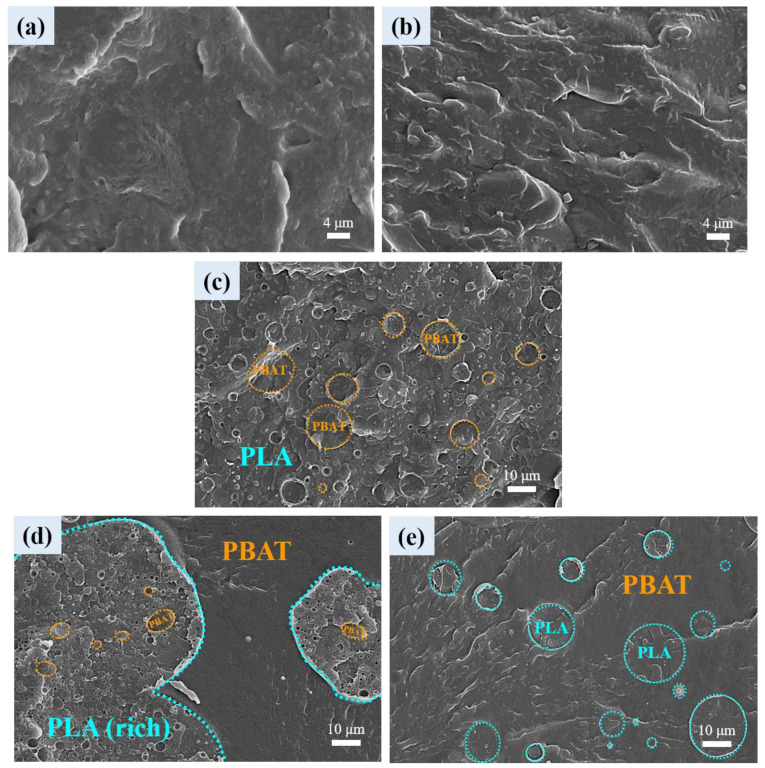
SEM images of (**a**) PLA, (**b**) PBAT, (**c**) L3B1, (**d**) L1B1, and (**e**) L1B3.

Figure 1a,b reveal the SEM images of neat PLA and PBAT, respectively. Both components show the typical rough texture of crystalline polymers. The SEM images of the blends are shown in Figure 1c–e, which exhibited bi-phasic morphology of immiscible characteristics. L3B1 and L1B3 (Figure 1c,e) exhibited sea-island morphology, with rounded minor component domains dispersed in the major component matrix. The dispersed domains had a broad distribution of diameter in the range of ca. 1–16 μm. Of the two blends, L1B3 had an average domain size of PLA larger than that of PBAT domains in L3B1 because of the higher viscosity of PLA than that of PBAT under the mixing condition (PLA showed evidently a higher melting temperature than that of PBAT). Regarding L1B1 (Figure 1d), a co-continuous-like morphology was constructed by PBAT continuous phase and PLA(rich) continuous phase with small PBAT domain inclusion. The morphology of the PLA(rich) phase was similar to that of L3B1 but had a smaller PBAT domain size.

**Figure 2:**
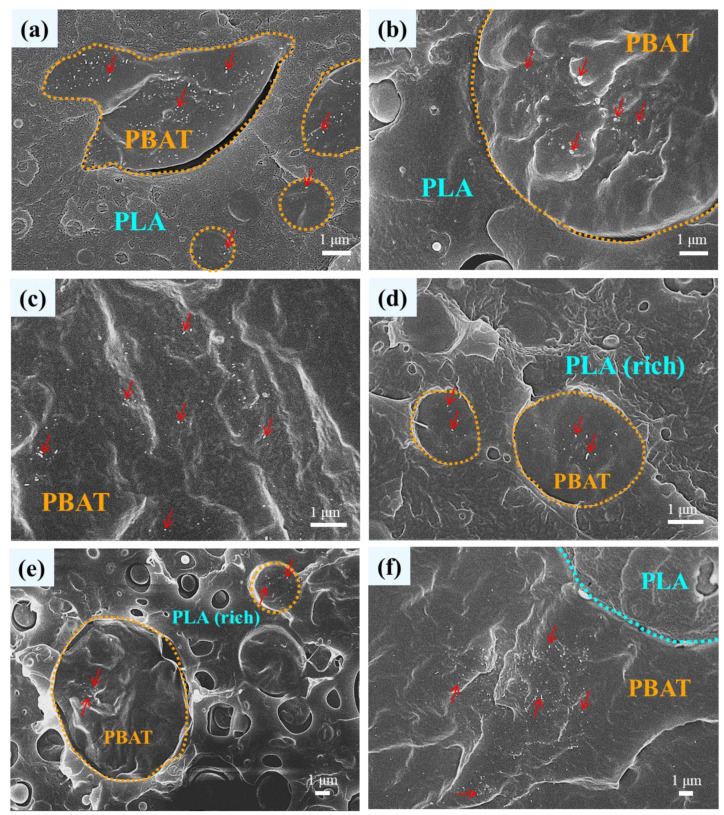
SEM micrographs of: (**a**) L3B1-T1, (**b**) L3B1-B1, (**c**) L1B1-T1(PBAT phase), (**d**) L1B1-T1(PLA-rich phase), (**e**) L1B1-B1(PLA-rich phase), and (**f**) L1B3-T1.

Figure 2 shows the high-magnification SEM images of representative blend-based composites with 1 phr CNTs or CBs inclusion to observe the dispersibility of added nanofillers. In L3B1-T1 and L3B1-C1 (Figure 2a,b), CNTs and CBs (arrowed) were selectively distributed in the dispersed PBAT domains (circled), which were hardly detected in the PLA matrix. The selective localization of CNTs in the PBAT phase of immiscible blends was reported previously [24], suggesting the superior affinity (π electron) of carbon nanofillers to PBAT. For L1B1-T1 (Figure 2c,d), the added CNTs were likewise localized in the PBAT continuous phase and the dispersed PBAT domains within the PLA(rich) continuous phase. The dispersibility of CBs in the PBAT domains within the PLA(rich) continuous phase of L1B1-B1 and CNTs in the PBAT matrix of L1B3-T1 are illustrated in Figure 2e,f, respectively. Based on the SEM results of Figure 1 and Figure 2, the L1B1 blend exhibited a (quasi)co-continuous phase morphology constructed by individual continuous PLA(rich) and PBAT phases, and the selective localization of carbon nanofillers in the PBAT phase of the nanocomposites was achieved.

### 3.2. Crystallization and Melting Behavior of Unfoamed Samples

**Figure 3:**
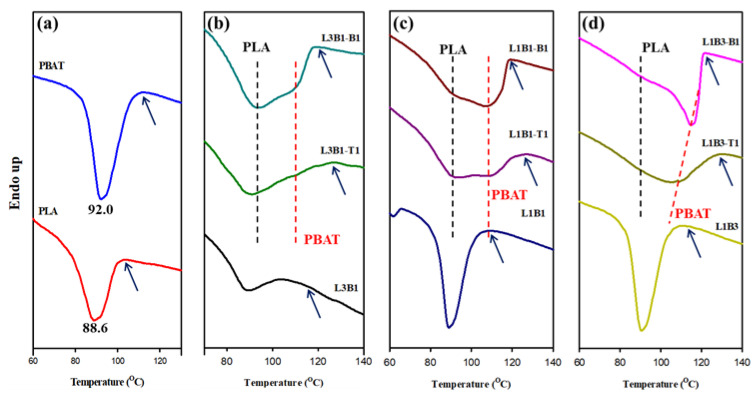
DSC 10 °C/min-cooled curves of (**a**) neat PLA and PBAT; (**b**) L3B1 and related composites; (**c**) L1B1 and related composites; and (**d**) L1B3 and related composites.

The effects of forming blends and composites on the crystallization of PLA and PBAT were evaluated. Figure 3 depicts the DSC curves of various samples, which were 10 °C/min-cooled from the molten state. Figure 3a denotes the crystallization peak temperatures (T_Lc_ and T_Bc_) of neat PLA and neat PBAT, which were 88.6 °C and 92.0 °C, respectively. For the three blends shown in Figure 3b–d, only one exothermic peak was displayed, which seemed to be the superposition of the individual crystallization of PLA and PBAT. The exothermic enthalpy varied with the composition of the blend, showing a higher value with increasing PBAT content. A limited effect was observed on the crystallization of PLA and PBAT after forming the immiscible blends. Regarding the CNT- or CB-added composites, the merged single exotherm in the corresponding blends evolved into partially overlapped exotherms, which had a high-temperature exotherm. The intensity of the high-temperature exotherm increased with increasing PBAT content, demonstrating that the exotherm was associated with the crystallization of PBAT. The appearance of the high-temperature PBAT exotherm clearly indicated the nucleation effect of CNT/CB on PBAT crystallization, resulting from the selective localization of CNTs or CBs in the PBAT phase. The nucleation effect of CNTs for PLA and PBAT was previously reported [14,33]. The initial crystallization temperature (T_i_, arrowed) in CNT-added composites was higher than that in corresponding CB-added composites, suggesting that CNT had a superior heterogeneous nucleation effect to CB on facilitating PBAT crystallization. The T_i_ of neat PBAT evidently increased from 114.3 °C to 128.3 °C in L1B3-T1. Regarding the crystallization temperature (peak position) of PLA, it did not vary after the addition of CNTs or CBs. CNTs and CBs, which were hardly localized in the PLA phase, were responsible for the observation. The differences in aspect ratio and surface structure were the main factors for the different efficiencies of CNT and CB in facilitating PBAT crystallization. The measurable T_i_, T_Lc_, and T_Bc_, with some estimated values from the partially overlapped exotherms of different samples, are compared in Table 2.

**Figure 4:**
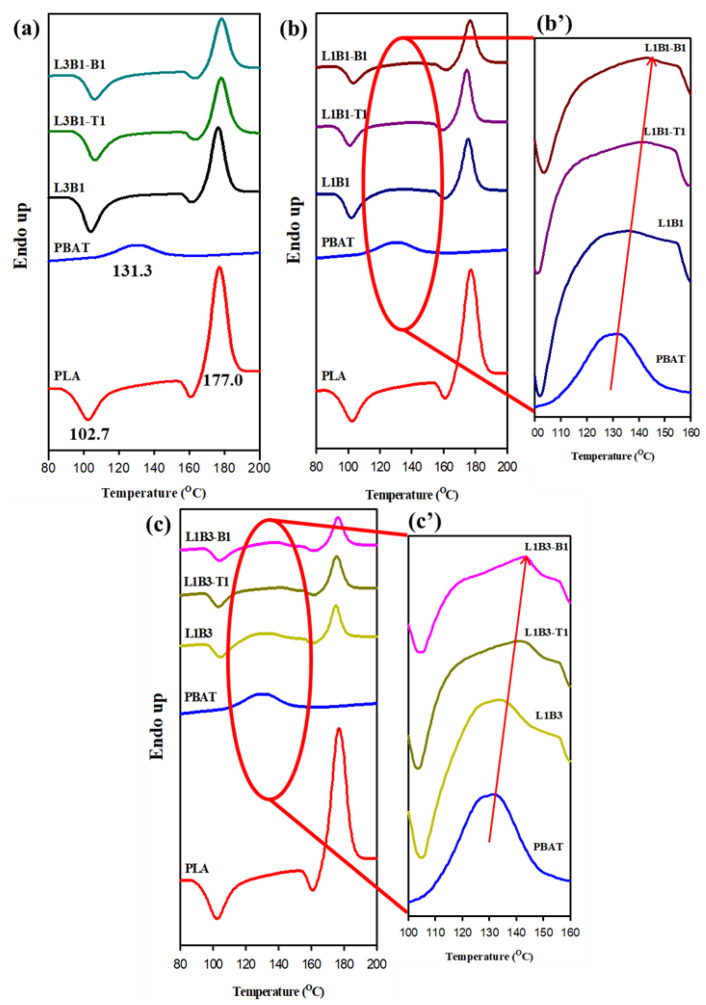
DSC 20 °C/min-heating curves of different samples after 10 °C/min pre-cooling: neat components and (**a**) L3B1 system, (**b**,**b’**) L1B1 system, and (**c**,**c’**) L1B3 system.

Figure 4 illustrates the DSC heating curves of 10 °C/min pre-cooled samples for evaluating the melting behavior of PLA and PBAT in different samples. In Figure 4a, neat PBAT exhibited a melting peak temperature (T_Bm_) at 131.3 °C, and neat PLA showed a cold crystallization peak and melting peak temperature (T_Lm_) at 102.7 °C and 177.0 °C, respectively. The typical shallow exotherm prior to PLA melting was due to the melting-recrystallization (annealing) of originally grown PLA crystals. For L3B1 and associated composites, the cold crystallization and melting of PLA were similar to those of neat PLA, whereas the melting of PBAT was hardly detected because of its low content. A slight increase in T_Lm_ was observed in the composites. Regarding L1B1, L1B3, and their associated composites (Figure 4b,c), the cold crystallization and melting of PLA again showed up, and the exothermic/endothermic enthalpy decreased as the PLA content decreased. The weak melting endotherm of PBAT was detected in L1B1- and L1B3-associated samples (Figure 4b’,c’), and the endotherm increased with increasing PBAT content. The determined T_Lm_ and T_Bm_ in different samples are summarized in Table 2. T_Bm_ increased after forming the blend and further increased after adding either CNTs or CBs. The existence of PLA crystals and the added CNTs/CBs should have induced more stable PBAT crystals grown during pre-cooling. The increased T_Bm_s in composites could be attributed to the decline in entropy change during crystal melting, thereby confining the effect caused by the dispersed CNTs or CBs.

### 3.3. Thermal Stability of Unfoamed Samples

**Figure 5:**
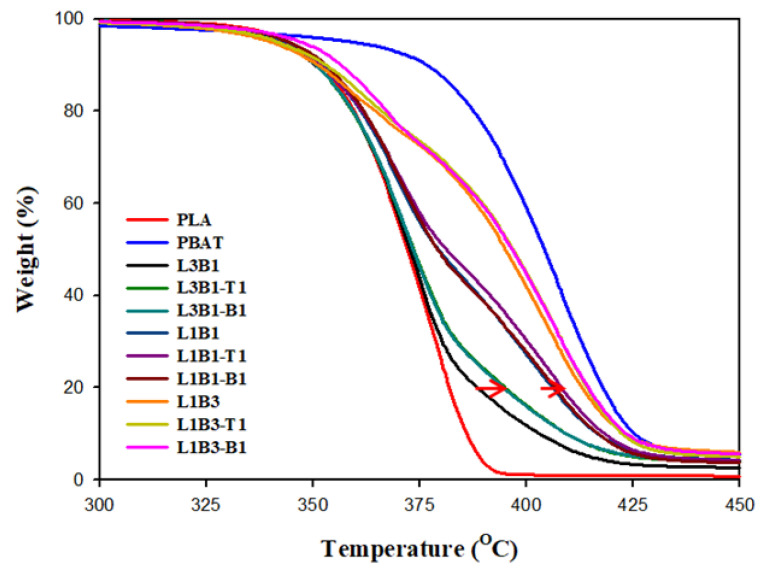
TGA curves of different samples heated at 10 °C/min in N_2_.

The TGA-scanned curves of the samples are presented in Figure 5. PBAT exhibited thermal stability (degradation temperature) that was evidently higher than PLA, mainly due to its aromatic feature. The blends and composites exhibited two-staged degradation, corresponding to the degradation of individual PLA and PBAT. A higher content of PBAT in the blends/composites shifted the curves close to that of neat PBAT and led to a more evident second-stage PBAT degradation. The presence of CNTs or CBs hardly modified the first stage (PLA portion) degradation in the composites, whereas the second stage (PBAT portion) degradation discernibly shifted to higher temperatures in L3B1- and L1B1-based composites (arrowed). The stability improvement in the second stage degradation was attributed to the dispersion of CNTs or CBs in the PBAT phase. Behera et al. [33] reported that the thermal stability of the PBAT portion of the CNT-added composites was improved compared with that of the PBAT/HDPE blend. The excellent heat stability and radical scavenging function of carbon fillers are responsible for the observation. The L1B3-based composites hardly exhibited thermal stability improvement, which could be attributed to the lower dispersion density of CNT/CB in the PBAT phase compared with those of the corresponding L3B1- and L1B1-based composites. The degradation temperatures (T_d20_ and T_d80_) at 20% and 80% weight loss (representative degradation of PLA and PBAT, respectively) are compared in Table 3. T_d80_ increased to 6 °C in L3B1-based composites compared with that in L3B1.

### 3.4. Mechanical Property of Unfoamed Samples

The tensile modulus (TM), tensile strength (TS), and elongation at break (EB) of the unfoamed samples were summarized in Table 3. Figure S1 depicts the typical stress-strain curves of the unfoamed samples. Neat PLA showed a TM value evidently higher than that of neat PBAT. TM of L3B1 and L1B1 were slightly higher than those (1741 MPa and 1196 MPa) calculated by the additivity rule. The (co)continuous phase played by PLA in L3B1/L1B1was responsible for the observation. L1B3 showed a TM much lower than the additivity rule calculated value (650 MPa) due to its immiscibility and the soft PBAT matrix. The TM values were adversely affected after adding CNTs or CBs in L3B1 and L1B1, which might be ascribed to the morphology modification and the location of CNTs/CBs in the PBAT domains. The L1B3-based composites, however, showed a slightly higher TM compared with L1B3 due to the localization of CNTs/CBs in the PBAT matrix. Regarding the TS of the samples, a similar formulation-dependent trend to that of TM results is observed. With increasing PLA content in the blends, TS gradually increased due to the higher TS of PLA compared with PBAT. However, the TS of the blends generally decreased after the loading of CNTs or CBs. Regarding the EB, PBAT had an evidently higher value than PLA. L3B1 and L1B1 exhibited lower EB values than those of neat components due to their immiscibility. L1B3 showed an EB slightly higher than that of PLA, attributed to the PBAT-dominated matrix. EB of the blends generally decreased after adding the CNTs or CBs.

### 3.5. Electrical Conductivity of Unfoamed Samples

The surface electrical resistivity (ER) of the prepared samples is compared in Table 3. The ER values of neat components and the blends were higher than 10^14^ Ω-cm, demonstrating their insulating nature. For L3B1-T1 and L3B1-B1, ER remained higher than 10^14^ Ω-cm because the CNT- or CB-included PBAT phase played the dispersed domains surrounded by the insulating PLA phase. The percolation of CNTs or CBs was not achieved without forming the continuous PBAT phase (network). ER dropped more than six orders of magnitude for the L1B1- and L1B3-based composites. Jen et al. [25] reported that the loading of 3 phr CNTs reduced the ER by eight orders of magnitude for PLA/TPEE blend-based composites. The drastic ER drop suggested the formation of (quasi)connected CNT or CB network in the continuous PBAT phase of the composites. The continuous PBAT phase is revealed in Figure 1 and Figure 2. (SEM images). The L1B1-based composites showed ER values slightly lower than the L1B3-based counterparts, attributed to the higher CNT or CB dispersion density in the PBAT phase of the L1B1 matrix than in the L1B3 matrix. After comparing the efficiency of adding CNTs and CBs in decreasing ER for the blend matrix, similar efficiency was obtained, though CNT had a higher aspect ratio than CB. The dispersibility and carbon purity of CNTs and CBs also played roles in affecting the electrical properties of the prepared composites.

### 3.6. Cellular Structure of Foamed Samples

**Figure 6:**
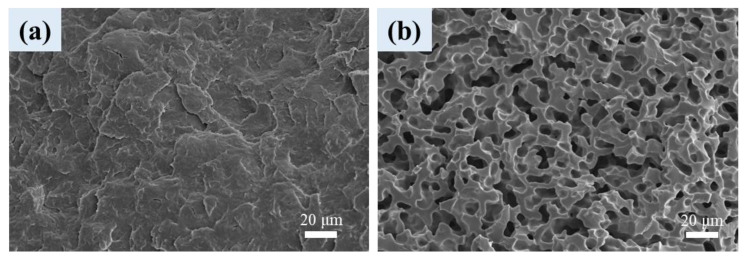
SEM images of scCO_2_-treated (**a**) PLA and (**b**) PBAT.

Figure 6 shows the internal structure of scCO_2_-treated neat PLA and PBAT, as revealed by SEM. After the treatment at 100 °C/4000 psi for 2 h, PLA hardly exhibited cellular structure, whereas PBAT showed cellular structure of irregular/elongated shape. The cell rupture/collapse in PBAT was ascribed to its low melt strength of PBAT. PLA hardly grew cellular structure under the treated condition, so the prospective selective foaming of the PBAT portion in the phase-separated blends/composites was preliminarily assessed.

**Figure 7:**
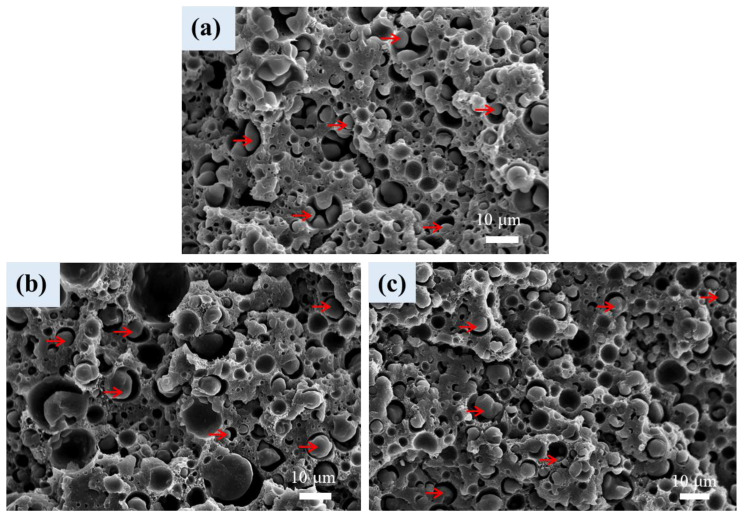
SEM cellular structure of (**a**) L3B1, (**b**) L3B1-T1, and (**c**) L3B1-B1.

Figure 7 shows the SEM internal structure of scCO_2_-treated (foamed) L3B1 and L3B1-based composites. L3B1 (Figure 7a) showed a partially foamed structure with the size of the closed cell correlated with the dispersed PBAT domains in the unfoamed counterpart (Figure 1), indicating the occurrence of selective foaming in the PBAT phase. The rounded cells of various sizes were finely distributed in the PLA matrix. The presence of an unfoamable PLA matrix confined PBAT in small domains and inhibited the collapse/rupture of PBAT cells during foaming. Some large-sized cells were formed around the boundary between the PBAT and PLA phases. Some PBAT particles (arrowed) existed within the large-sized cells. The weak adhesion between PLA-PBAT phases led to cell nucleation and growth along the boundary. For the scCO_2_-treated L3B1-based composites, a cellular structure similar to that of foamed L3B1 was observed (Figure 7b,c). The average size of PBAT cells in the composites was slightly larger than that in foamed L3B1, which might be attributed to the CNT/CB-caused aggregation in original PBAT domains prior to the scCO_2_-treatment.

**Figure 8:**
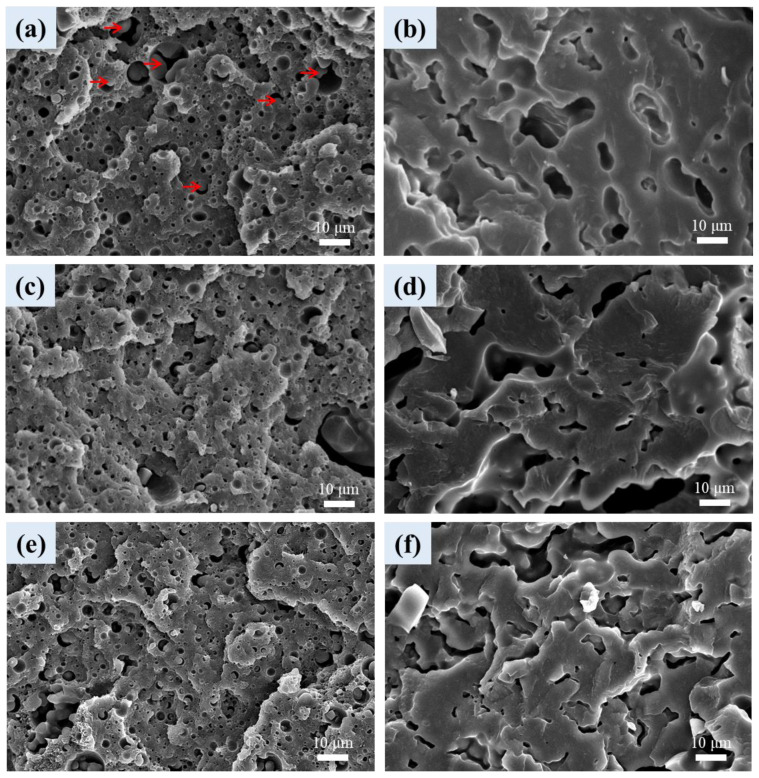
SEM cellular structure of (**a**) PLA(rich) phase in L1B1, (**b**) continuous PBAT phase in L1B1, (**c**) PLA(rich) phase in L1B1-T1, (**d**) continuous PBAT phase in L1B1-T1, (**e**) PLA(rich) phase in L1B1-C1, and (**f**) continuous PBAT phase in L1B1-C1.

As un-treated L1B1 exhibited (quasi)co-continuous PLA(rich) phase and PBAT phase (Figure 1), Figure 8a,b show the cellular structure in the individual PLA(rich) phase and PBAT phase of scCO_2_-treated L1B1. In the PLA(rich) phase of Figure 8a, sphere-like PBAT cells with size/shape similar to the original un-foamed PBAT domains were developed. Large PBAT cells with particle (arrowed) inclusion were also observed. Figure 8b shows the irregular/elongated shaped PBAT cells in the PBAT continuous phase, similar to those observed in scCO_2_-treated neat PBAT. Figure 8c–f depicts the PBAT cellular structure in the individual PLA(rich) and PBAT continuous phases of L1B1-T1 and L1B1-C1 composites, respectively. The cellular structure was similar to those developed in the PLA(rich) phase and PBAT phase of parent L1B1 foam. The average PBAT cell size in the PLA(rich) phase was smaller in the composites compared with that in parent L1B1 foam, which was ascribed to CNT/CB-induced cell nucleation.

**Figure 9:**
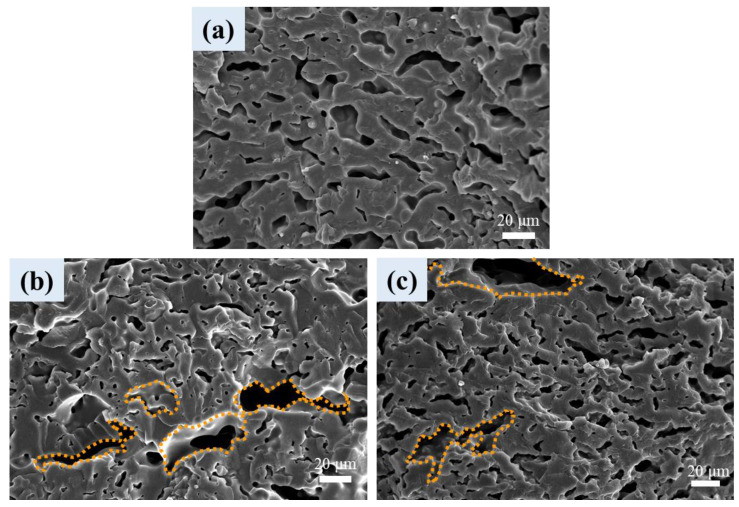
SEM cellular structure of PBAT phase in (**a**) L1B3, (**b**) L1B3-T1, and (**c**) L1B3-C1.

For the L1B3 blend and composites of Figure 9, irregular/elongated shaped cells in the PBAT matrix were observed. The cells in composites (Figure 9b,c) were generally smaller than those observed in parent L1B3 foam (Figure 9a), which was again caused by the CNT- or CB-induced cell nucleation during foaming. The cell density was higher in the composites compared with their parent blend. A few large cells (not nucleated by the CNTs/CBs) also showed up in the PBAT matrix of the composites (surrounded by a dashed line). The average cell size and porosity of the foamed samples are summarized in Table 3. In this regard, techniques such as improving the miscibility between PLA and PBAT or inducing the branching/crosslinking of PBAT chains in samples are feasible to modify the irregularities of the PBAT cell structure.

### 3.7. Melting Behavior of Foamed Samples

**Figure 10:**
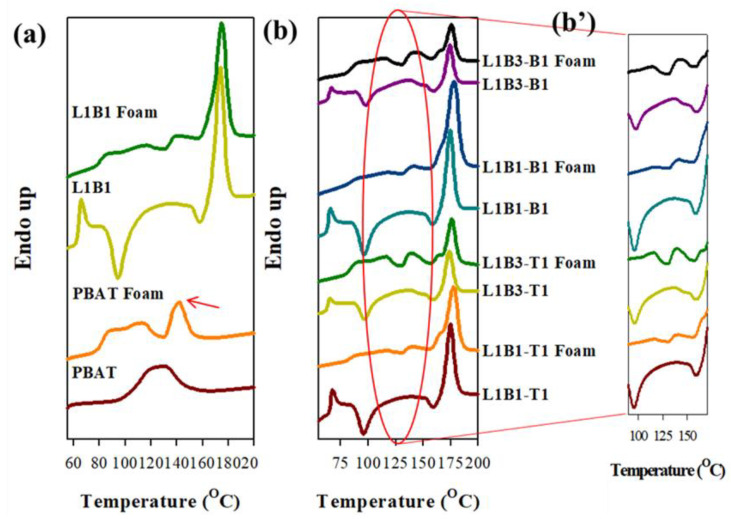
DSC 20 °C/min-heating curves of samples w/o scCO_2_-treatment (foaming): (**a**) PBAT and L1B1 and (**b,b’**) L1B1- and L1B3-based composites.

Foaming selectively occurred in the PBAT phase of the samples. The DSC melting behavior of PBAT in the scCO_2_-treated/untreated representative samples is compared in Figure 10. The results of the L3B1 system were not shown due to the low content of PBAT. Figure 10a compares the heating curves of scCO_2_-treated and untreated PBAT. The original single broad endotherm of untreated PBAT evolved into a low-temperature broad endotherm (ca. 80–120 °C) and a high-temperature melting peak (arrowed, around 142 °C) after the scCO_2_ treatment. The melting behavior modification indicates that the scCO_2_ treatment must have aligned some PBAT chains in a perfect/thick lamellar crystal state and also induced some thin lamellar crystals, leading to the stability of the bimodal crystals. The heating curves of treated/untreated L1B1 are depicted in Figure 10a. Similar to neat PBAT, high-temperature, and low-temperature melting endotherms of PBAT crystals were observed in foamed L1B1. In addition, the PLA cold crystallization, which existed in untreated L1B1, was not observed after the scCO_2_ treatment and showed a slightly higher temperature (T_Lm_) melting peak of PLA compared with the untreated counterpart. These observations demonstrated that PLA cold crystallization was facilitated and occurred during the scCO_2_ treatment, and more perfect PLA crystals were grown compared with the cold-crystallized PLA crystals in the untreated sample. For the representative scCO_2_-treated L1B1- and L1B3-based composites, the modification in PBAT melting behavior similar to those of treated PBAT and L1B1 was also observed (Figure 10b). The melting endotherm of PBAT increased as its content increased in the composites. The disappearance of PLA cold crystallization and the rise in PLA melting temperature were again observed in the treated composites.

### 3.8. Hardness of Unfoamed/Foamed Samples

The rigidity-associated hardness of the prepared samples was measured, and the values are recorded in Table 4. Neat PLA possessed a higher hardness than that of neat PBAT (82 vs. 51), confirming the more rigid characteristic of PLA. The blend with higher PLA content possessed a higher hardness value. The addition of 1 phr rigid CNTs or CBs slightly increased the hardness of the parent blends. Regarding the foamed samples, the hardness declined compared with their unfoamed counterparts. The formation of cellular structure in the PBAT phase was responsible for the decrease in hardness.

## 4. Conclusions

In this study, a twin-screw extruder was used to fabricate PLA/PBAT blends and blend-based nanocomposites with CNT or CB inclusion. Neat components and prepared blends/composites were further treated with scCO_2_ to prepare samples selectively foamed in the PBAT phase. SEM results confirmed the immiscible characteristic of the blends, and a (quasi)co-continuous phase morphology was developed in the L1B1 blend. The added CNTs or CBs were selectively distributed in the PBAT phase of the blend-based composites and, thus, played as nucleation agents for PBAT crystallization in different composites. The thermal stability of the PBAT portion in different composites was slightly improved due to the selective dispersion of CNTs/CBs in the PBAT phase. Compared with the parent blends, the electrical resistivity decreased by more than six orders of magnitude for L1B1- and L1B3-based composites, confirming the formation of double percolation phase morphology of PBAT-CNT/CB phase in the composites. After the scCO_2_ treatment under a designed condition, selective foaming in the PBAT phase of the blends and composites was observed. The cellular structure of the PBAT phase in the blends was similar to the size/shape of PBAT domains in the untreated blends or the cellular structure in the neat PBAT foam, which suggested the PLA counterpart was hardly involved in the foaming process. The presence of CNTs or CBs in the composites slightly reduced the cell size of the foamed PBAT phase due to the CNT/CB-induced cell nucleation. The scCO_2_ treatment induced the growth of the bimodal stability of PBAT crystals (two groups of lamellar thickness) in different samples, and PBAT melted mainly in two temperature regions. The hardness of the blends increased with increasing PLA content and slightly increased after forming the rigid CNT/CB-added corresponding composites. The foamed samples showed lower hardness than the corresponding unfoamed samples.

## Figures and Tables

**Table 1:** Sample codes and formulation.

Sample Code	Composition	Parts (wt.%)
PLA	PLA	100
PBAT	PBAT	100
L3B1	PLA/PBAT	75/25
L1B1	PLA/PBAT	50/50
L1B3	PLA/PBAT	25/75
L3B1-T1	PLA/PBAT/CNT	75/25/1 *
L1B1-T1	PLA/PBAT/CNT	50/50/1 *
L1B3-T1	PLA/PBAT/CNT	25/75/1 *
L3B1-B1	PLA/PBAT/CB	75/25/1 *
L1B1-B1	PLA/PBAT/CB	50/50/1 *
L1B3-B1	PLA/PBAT/CB	25/75/1 *

* phr: parts per hundred parts of resin.

**Table 2:** DSC data of representative samples.

Samples	Properties				
	T_i_ (°C)	T_Lc_ (°C)	T_Bc_ (°C)	T_Lm_ (°C)	T_Bm_ (°C)
PLA	105.0	88.6	-	177.0	-
PBAT	114.3	-	92.0	-	131.3
L3B1	107.9	88.5	-	176.3	
L3B1-T1	127.0	91.2	-	178.0	
L3B1-B1	122.3	93.5	109.0	178.0	
L1B1	112.0	88.8	-	175.2	136.2
L1B1-T1	126.6	92.2	107.9	174.6	141.8
L1B1-B1	119.5	89.5	107.2	176.6	143.3
L1B3	115.0	90.0	-	175.0	133.7
L1B3-T1	128.3	-	107.6	175.5	141.6
L1B3-B1	122.2	-	115.0	176.5	143.0

**Table 3:** TGA data/tensile test data/electrical resistivity of unfoamed samples and cell size/porosity of foamed samples.

Samples				Properties				
	T_20%_ (°C)	T_80%_ (°C)	TM (MPa)	TS (MPa)	EB (%)	Log ER (Ω-cm)	Cell Size (μm)	Porosity
PLA	360	382	2287 ± 49	61.5 ± 0.7	3.3 ± 0.1	>14	-	-
PBAT	388	418	105 ± 2	5.6 ± 0.4	9.2 ± 1.3	>14	7.76 ± 2.12	0.48
L3B1	359	389	1812 ± 44	43.7 ± 1.1	3.1 ± 0.1	>14	2.72 ± 1.27	0.19
L3B1-T1	359	395	1687 ± 39	32.2 ± 1.3	2.6 ± 0.2	>14	3.44 ± 1.37	0.26
L3B1-B1	359	395	1662 ± 40	35.4 ± 1.2	2.5 ± 0.2	>14	2.98 ± 1.26	0.24
L1B1	361	406	1025 ± 55	13.1 ± 1.1	2.0 ± 0.3	>14	0.84 ± 0.45 *	0.22
L1B1-T1	362	409	802 ± 21	9.3 ± 1.2	1.9 ± 0.2	7.7 ± 0.5	0.53 ± 0.34 *	0.19
L1B1-B1	362	408	848 ± 38	8.9 ± 1.1	1.7 ± 0.2	7.8 ± 0.3	0.51 ± 0.35 *	0.18
L1B3	365	413	221 ± 5	7.5 ± 0.6	4.8 ± 0.5	>14	5.25 ± 4.18	0.32
L1B3-T1	366	414	229 ± 5	7.2 ± 0.7	5.0 ± 0.5	9.0 ± 0.7	3.32 ± 2.13	0.26
L1B3-B1	367	415	244 ± 15	5.7 ± 0.4	4.0 ± 0.3	8.2 ± 0.2	4.30 ± 1.66	0.24

* in PLA(rich) phase.

**Table 4:** Hardness data of prepared samples.

	Samples					
	PLA	PBAT	PBAT Foam			
Hardness	82	51	40			
	L3B1	L3B1 foam	L3B1-T1	L3B1-T1 foam	L3B1-B1	L3B1-B1 foam
Hardness	71	60	73	61	72	63
	L1B1	L1B1 foam	L1B1-T1	L1B1-T1 foam	L1B1-B1	L1B1-B1 foam
Hardness	61	53	62	55	63	58
	L1B3	L1B3 foam	L1B3-T1	L1B3-T1 foam	L1B3-B1	L1B3-B1 foam
Hardness	53	41	55	43	54	43

## Data Availability

The data presented in this study are available on request.

## Supplementary Materials

The following supporting information can be downloaded at: https://www.mdpi.com/article/10.3390/polym16141971/s1, Figure S1: Typical stress-strain curves of unfoamed samples.
