# Supplementary material for: Fabrication and Characterization of PLA/PBAT Blends, Blend-Based Nanocomposites, and Their Supercritical Carbon Dioxide-Induced Foams
Source: Polymers (Basel). 2024 Jul 10;16(14):1971. doi: 10.3390/polym16141971 (PMC11281301; doi:10.3390/polym16141971)
Supplement: Supplementary file 1 [file polymers-16-01971-s001.zip › polymers-3021851-supplementary.pdf]

Supplementary Materials

Fabrication and Characterization of PLA/PBAT Blends, Blend-Based Nanocomposites, and Their Supercritical Carbon Dioxide-Induced Foams

Kartik Behera [1], Chien-Hsing Tsai [1], Xiang-Bo Liao [1] and Fang-Chyou Chiu [1,2,3,*]

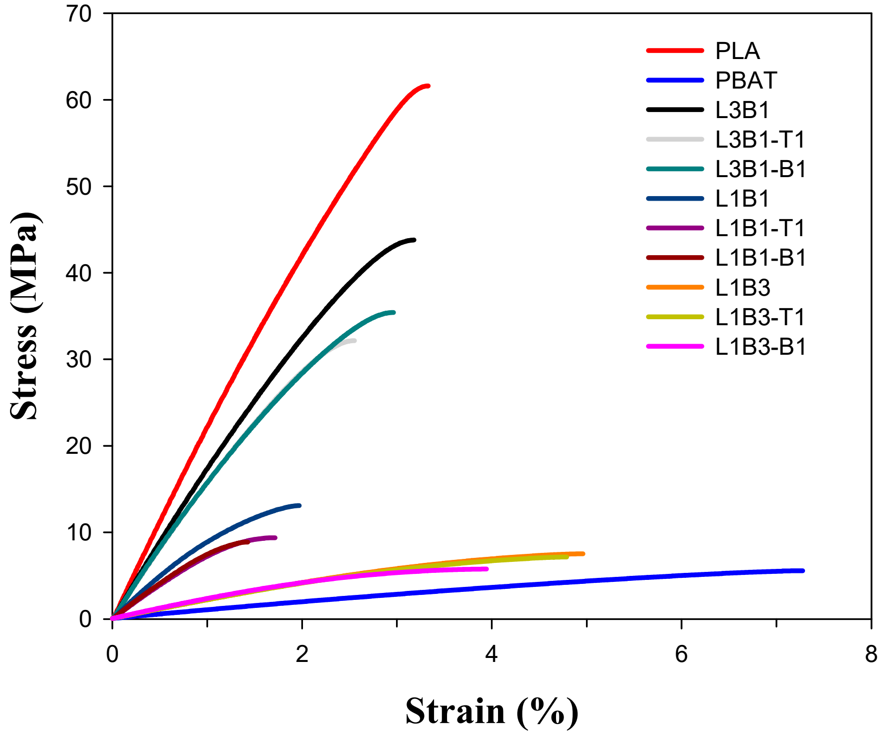

Figure S1. Typical stress-strain curves of unfoamed samples.
